# Development of Oral Care Chip, a novel device for quantitative detection of the oral microbiota associated with periodontal disease

**DOI:** 10.1371/journal.pone.0229485

**Published:** 2020-02-28

**Authors:** Ai Nozawa, Hiroyuki Oshima, Naoyuki Togawa, Takenori Nozaki, Shinya Murakami

**Affiliations:** 1 Tsurumi R&D center, Mitsubishi Chemical Corporation, Yokohama, Kanagawa, Japan; 2 Division of Interdisciplinary Dentistry, Osaka University Dental Hospital, Suita, Osaka, Japan; 3 Department of Periodontology, Osaka University Graduate School of Dentistry, Suita, Osaka, Japan; New York Medical College, UNITED STATES

## Abstract

Periodontal disease, the most prevalent infectious disease in the world, is caused by biofilms formed in periodontal pockets. No specific bacterial species that can cause periodontitis alone has been found in any study to date. Several periodontopathic bacteria are associated with the progress of periodontal disease. Consequently, it is hypothesized that dysbiosis of subgingival microbiota may be a cause of periodontal disease. This study aimed to investigate the relationship between the subgingival microbiota and the clinical status of periodontal pockets in a quantitative and clinically applicable way with the newly developed Oral Care Chip. The Oral Care Chip is a DNA microarray tool with improved quantitative performance, that can be used in combination with competitive PCR to quantitatively detect 17 species of subgingival bacteria. Cluster analysis based on the similarity of each bacterial quantity was performed on 204 subgingival plaque samples collected from periodontitis patients and healthy volunteers. A significant difference in the number of total bacteria, *Treponema denticola*, *Campylobacter rectus*, *Fusobacterium nucleatum*, and *Streptococcus intermedia* bacteria in any combination of the three clusters indicated that these bacteria gradually increased in number from the stage before the pocket depth deepened. Conversely, *Porphyromonas gingivalis*, *Tannerella forsythia*, *Prevotella intermedia*, and *Streptococcus constellatus*, which had significant differences only in limited clusters, were thought to increase in number as the pocket depth deepened, after periodontal pocket formation. Furthermore, in clusters where healthy or mild periodontal disease sites were classified, there was no statistically significant difference in pocket depth, but the number of bacteria gradually increased from the stage before the pocket depth increased. This means that quantitative changes in these bacteria can be a predictor of the progress of periodontal tissue destruction, and this novel microbiological test using the Oral Care Chip could be effective at detecting dysbiosis.

## Introduction

Periodontal disease is an infectious disease caused by oral bacteria that inhabit biofilms formed in the subgingival pocket. It is known that bacterial species forming subgingival plaques are grouped into several microbial complexes [1]. It is hypothesized that the complex composed of *Porphyromonas gingivalis*, *Tannerella forsythia*, and *Treponema denticola* is responsible for the initiation and progress of periodontal disease since these bacterial species are frequently isolated from severe periodontal lesions [2]. However, a meta-analysis report has shown that *P*. *gingivalis* is not always found in all subgingival microbiotas of deep periodontal pockets [3]. In addition, no specific bacterial species that can cause periodontitis alone, has been found in any animal model study to date. Therefore, a hypothesis is proposed that periodontal disease is not caused by several specific bacterial species, but by the interactions between the host and the dysbiotic subgingival microbiota [4]. Existing methods for analysis of microbiota data is not quantitative or clinically applicable, although detecting these specific bacteria is a key tool for the diagnosis of periodontal disease and assessing treatment effectiveness.

This study aimed to investigate the relationship between the subgingival microbiota and clinical findings of periodontal disease in a quantitative and clinically applicable way. The analysis procedure was performed using a large-scale sample cluster analysis containing healthy and periodontal disease sites, based on the similarity of the microbiota proportions, or by comparing the subgingival microbiota proportions before and after periodontal treatment.

Several detection methods using anaerobic culture, immunofluorescent antibodies, DNA probes, and polymerase-chain-reaction (PCR) have been developed [5, 6]. There have also been many reports using competitive PCR to detect bacteria quantitatively [7, 8]. However, it was previously difficult to accurately determine the number of each species of bacterium in the subgingival microbiota, because there were several technical difficulties associated with methods that detect multiple targets [9]. Therefore, by applying the recently developed methods with an improved quantitative performance by combining microarray and competitive PCR [10], more bacterial species can be explored in this study. Oral Care Chip is a new device, which was developed to provide a simultaneous and quantitative analysis of 17 subgingival bacteria to acquire microbiota data.

## Materials and methods

### Oral Care Chip

We first developed a novel DNA microarray Oral Care Chip containing DNA probes to measure the total number of bacteria and detect 17 species of specific bacteria assumed to be responsible for the initiation and progress of periodontal disease [1, 2]. The sequences of the DNA probes for determining the total number of bacteria were designed according to sequences in the conserved region of V3 of 16S rRNA; the sequences of specific probes for each bacterial species were selected among sequences in the specific V3 region of 16S rRNA based on the National Center for Biotechnology Information (NCBI) database (National Library of Medicine, Bethesda, MD, USA) (Table 1). The specificity and hybridization efficiency of each probe on the Oral Care Chip were confirmed individually. In this process, it became clear that the probes for *Fusobacterium nucleatum subspecies animalis* and *F*. *nucleatum subsp*. *nucleatum* (probe no.06 and no.07) hybridized with each other, because the DNA sequence of these subspecies have significant similarity.

**Table 1:** The sequences of probes on Oral Care Chip.

Probe no.	Probe	Sequence (5′–3′)	Accession no.	Nucleotide location^a^	16S rRNA copies	Hybridization coefficient
01	Total number of bacteria	CGTATTACCGCGGCTGCTGGCAC	–	–	4.5	–
02	*Porphyromonas gingivalis*	TTCAATGCAATACTCGTATC	AB035459	464	4	0.80
03	*Tannerella forsythia*	CACGTATCTCATTTTATTCCCCTGT	AP013044	442	2	4.4
04	*Treponema denticola*	CCTCTTCTTCTTATTCTTCATCTGC	AE017226	442	2	5.8
05	*Campylobacter rectus*	GTCATAATTCTTTCCCAAGA	ACFU01000050	436	3^b^	1.2
06	*Fusobacterium nucleatum subsp*. *animalis*	TTTCTTTCTTCCCAACTGAA	CP022124	437	4	0.68
07	*Fusobacterium nucleatum subsp*. *nucleatum*	TACATTCCGAAAAACGTCAT	AE009951	411	5	1.5
08	*Prevotella intermedia*	CGAAGGGTAAATGCAAAAAGGC	CP019300.1	477	4	1.2
		CGAAGGGTAAATGCAAAGGGGC				
09	*Prevotella nigrescens*	CTTTATTCCCACATAAAAGC	X73963	443	4^b^	0.68
10	*Streptococcus constellatus*	AAGTACCGTCACTGTGTG	CP003840	488	4	0.28
11	*Aggregatibacter actinomycetemcomitans*	GTCAATTTGGCATGCTATTAACACACC	CP001733	457	6	2.9
		GTCAAGTTGGCATGCTATTAACACACC	CP016553			
12	*Capnocytophaga gingivalis*	TACACGTACACCTTATTCTT	X67608	442	3	1.6
13	*Streptococcus gordonii*	CACCCGTTCTTCTCTTACA	AF003931	454	4	1.5
14	*Streptococcus intermedius*	ACAGTATGAACTTTCCATTCT	AP014880	475	4	1.9
15	*Veillonella parvula*	TCCTTCTAACTGTTCGC	LT906445	482	4	0.24
16	*Actinomyces viscosus*	CCACCCACAAGGAGCAG	X82453	459	3^b^	1.7
17	*Selenomonas noxia*	TTCGCATTAGGCACGTTC	AF287799	467	4^b^	0.36
18	Streptococci	TTAGCCGTCCCTTTCTGG	–	–	6^b^	1.0

Probes no. 08 and 11 constitute an equimolar mix of two different sequences.

^a^The first nucleotide in 16S rRNA to which the probe hybridizes.

^b^In the absence of appropriate information, the median value for the genus stated in the Ribosomal RNA Database version 5.5 was used.

The synthesized probes were next mounted onto a fibrous DNA chip platform Genopal^™^ (Mitsubishi Chemical, Tokyo, Japan) as previously described [11]. The probes were accordingly assigned to five spots on one microarray.

### Competitive PCR and hybridization

As the internal control for amplification, we synthesized an artificial oligonucleotide target mimic with a 463-bp sequence that hybridizes to control probe and PCR primer sets at both ends. Subsequently, it was ligated into the pUC19 vector (S1 Fig; S1 Table). Then, competitive PCR and hybridization were carried out in the following steps. Forward V3 forward primer (5′-Cy5-TACGGGAGGCAGCAG-3′) and V4 reverse primer (5′-TACCIGGGTATCTAATCC-3′) were used for competitive PCR. PCR was conducted using 0.5 amol of control DNA, 20 pmol of each primer, 10 μl of 2× PCR solution Premix Ex Taq^™^ Hot-start version (Takara, Shiga, Japan), and template (as described below), in a total volume of 20 μl. The reaction was started by an initial denaturation of 1 min at 95 °C, followed by 40 cycles of 10 s at 98 °C, 30 s at 55 °C, and 20 s at 72 °C. The amplicon length was approximately 440 bp. The PCR product was directly suspended in 180 μl of hybridization solution (48 μl of 1 M tris-HCl pH7.5, 48 μl of 1 M NaCl, 20 μl of 0.5% tween-20, and 64 μl of Milli-Q water), hybridized with the probes on the Oral Care Chip at 50 °C for 16 h, and washed with the Genopal^™^ instrument system (Mitsubishi Chemical). Hybridization signal intensity (SI) was determined using multi-beam excitation technology and Genopal reader (Mitsubishi Chemical). SI for subsequent analyses was obtained by deducting the SI median of background spots from the SI median of the five spots on each probe. The background spots were spots with no probe mounted therein. For each array, an SI median of the background spots + 3σ was treated as the detection limit value.

As PCR templates, MSA-1003^™^ containing mixed genomic material of 20 strains (American Type Culture Collection, Manassas, VA, USA), plasmid DNA, or subgingival plaque samples was used.

### Quantitative detection of 17 species of oral bacteria

As the first step of quantitative detection, we measured the total amount of 16S rRNA using the standard calibration curve plotted in reference to a previous method [10]. Next, we determined the number of each bacterial species using each species-specific probe SI corrected with hybridization affinity ratio (Table 1, S3 Fig).

Data from the Ribosomal RNA Database version 5.5 (the Schmidt Laboratory at the University of Michigan, Ann Arbor, MI, USA) were used to determine the number of copies of 16S rRNA (Table 1). In the absence of appropriate information, the median value for the genus was used. To calculate the total number of bacteria in samples, 16S rRNA copy numbers relative to genomic DNA was assumed to be 4.5, calculated based on a weighted average reported in a study in which the predominant and prevalent bacterial species in the saliva of orally healthy subjects were determined by pyrosequencing [12]. The bacterial counts were calculated by multiplying the Avogadro’s constant based on the molecular weight of the genome (i.e. the molecular weight of 16S rRNA was divided by the number of 16S rRNA copies).

### Verification of the validity of Oral Care Chip

To verify the validity of Oral Care Chip with respect to representative 6 periodontopathic bacterial species, real-time PCR was performed using the 7500 Fast Real-time PCR System and TaqMan^™^ Fast Universal PCR Master Mix, no AmpErase^™^ UNG (Applied Biosystems, Foster City, CA, USA). There were 121 measurement target samples before and after SRP treatment. Three samples with insufficient residual volume for verification were excluded. The compositions of the reagents used were as specified by the instruction manual; 1 μl of template was analyzed. The experiments were performed under the following conditions: 20 s at 95 °C, followed by 40 cycles of 3 s at 95 °C, and 30 s at 60 °C for each bacterium, or followed by 40 cycles of 15 s at 95 °C and 60 s at 60 °C for the detection of the total number of bacteria. The probes and primers used were as described elsewhere [13–16], with the exception of those for *T*. *forsythia* for which 1 base at the 5′-end of the reverse primer was deleted because that particular base varied among the different strains (S2 Table). Similar to the Oral Care Chip probe, it was confirmed that the real-time PCR probe completely matched the sequence of the standard strain. Standard curves for each bacterium were generated, accordingly, using the following DNA samples: *P*. *gingivalis* ATCC^®^ 33277D-5, *T*. *forsythia* ATCC^®^ 43037 D-5, *T*. *denticola* ATCC^®^ 35405 D-5, *Prevotella intermedia* ATCC^®^ 25611 D-5, *Aggregatibacter actinomycetemcomitans* ATCC^®^ 700685 D-5, and ATCC^®^ MSA-1002 (American Type Culture Collection) for the total number of bacteria. The detection limit was determined to be a threshold of 35 cycles.

### Clinical samples

This study was conducted in accordance with the principles of the Declaration of Helsinki and was also approved by the ethics committee of Osaka University Graduate School of Dentistry (approval number: H20-E9). Prior to the selection of subjects, we explained the purpose of this study and possible disadvantages in detail both verbally and in writing, and then obtained written informed consent. A total of 64 patients with periodontal disease (25 males and 39 females; mean age, 47.9 ± 14.8 years) who visited the Osaka University Dental Hospital at first presentation and 72 healthy volunteers (46 males and 26 females; mean age, 25.7 ± 6.1 years) participated in this study (Table 2). We initially examined their periodontal tissue and recorded probing depth (PD), bleeding on probing (BOP), gingival index (GI), and plaque index (PlI) as clinical parameters. Two samples were taken from the patients with periodontal disease: one severely diseased site with deep periodontal pockets (PD ≥ 6 mm) and one moderately diseased site (4 mm ≤ PD < 6 mm) either in a neighboring tooth or in the contralateral tooth, respectively, for examination, whereas in the healthy volunteers, one healthy site with PD < 3 mm and GI < 1 was selected for plaque sampling. For these two patients, the above 2 sets and 4 samples were also collected. Among the patients with periodontal disease, we obtained samples from 31 patients who had agreed to sampling (total of 62 samples) after scaling and root planing (SRP). Samples were obtained from periodontal pockets with #40 absorbent points (Dentsply Maillefer, Ballaigues, Switzerland). Next, 200 μl of distilled water was added to the samples and vortex-mixed for 20 s. Then, the samples were stored at −80 °C. Prior to the analyses, the samples were pre-heated at 80 °C for 10 min, and 1 μl of 200-μl samples was used as a DNA template for PCR.

**Table 2:** Clinical parameters of participants.

	Patients with periodontal disease			Healthy volunteers		
Subjects	64			72		
Age	47.9 (14.8)			25.7 (6.1)		
Sex (male / female)	25 / 39			46 / 26		
Samples	132			72		
Pd (mm)^a^	5.9 (0.2)			2.6 (0.1)		
BOP (positive rate)^b^	65%			6%		
	0	1	2	0	1	2
GI (sites)^c^	3	80	49	71	1	0
plI (sites)^d^	11	83	38	52	19	1

The value in parentheses indicates standard error.

^a^ probing depth

^b^ bleeding on probing,

^c^ gingival index

^d^ plaque index

### Statistical analysis

All analyses were conducted using R version 3.1.5 (R Foundation for Statistical Computing, Vienna, Austria). To test the correlation between the Oral care chip and real-time PCR, the Pearson correlation coefficient was used. Similarities between microbiota were analyzed with the Ward’s method for clustering and the difference between PD and the numbers of each bacterial species in respective clusters was examined with the Steel-Dwass multiple comparison test. A Wilcoxon signed-rank test was used to determine changes in PD and the number of each bacterium before and after periodontal disease treatment. Among clinical information, BOP, GI, and plI were treated as categorical variables. The Chi-square test was used to compare three clusters, and the McNemar test was used for comparisons before and after treatment. The significance level was set to 0.05 for all tests.

## Results

### Evaluation of the quantitative performance of the Oral Care Chip

To produce the standard curves to calculate the total counts of bacteria, input/output ratios were plotted (S2 Fig). The SI obtained by MSA-1003^™^ evaluation was analyzed to calculate an output ratio of log [target probe SI (nW/m^2^)/control DNA probe SI (nW/m^2^)], and the amount of template DNA was analyzed to calculate an input ratio of log [target DNA (amol)/control DNA (amol)]. The spot images from the Oral Care Chip are shown in S2 Fig.

**Fig 1:**
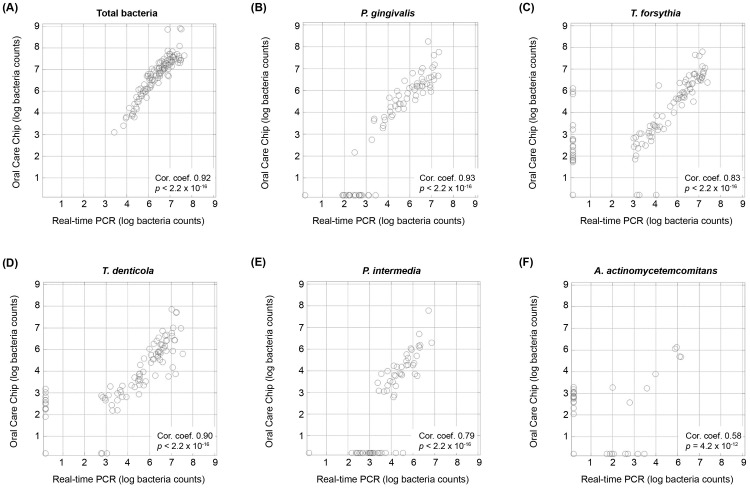
Comparison of the performance of Oral Care Chip and real-time PCR. The plots show bacterial counts per one paper point, expressed as log10 values. The values below the detection limit were replaced with 0.1. Cor. coef. means Pearson correlation coefficient. (A) Total number of bacteria; (B) *Porphyromonas gingivalis*; (C) *Tannerella forsythia*; (D) *Treponema denticola*; (E) *Prevotella intermedia*; and (F) *Aggregatibacter actinomycetemcomitans*.

The data obtained by Oral Care Chip and real-time PCR were highly correlated, as indicated by the significant and low p-values (Fig 1). In contrast, some samples yielded different results with the two methods, probably because the probes had different specificities for different strains, except for the representative strains, as revealed by BLAST search analysis (NCBI).

### Cluster analysis of subgingival microbiota

**Fig 2:**
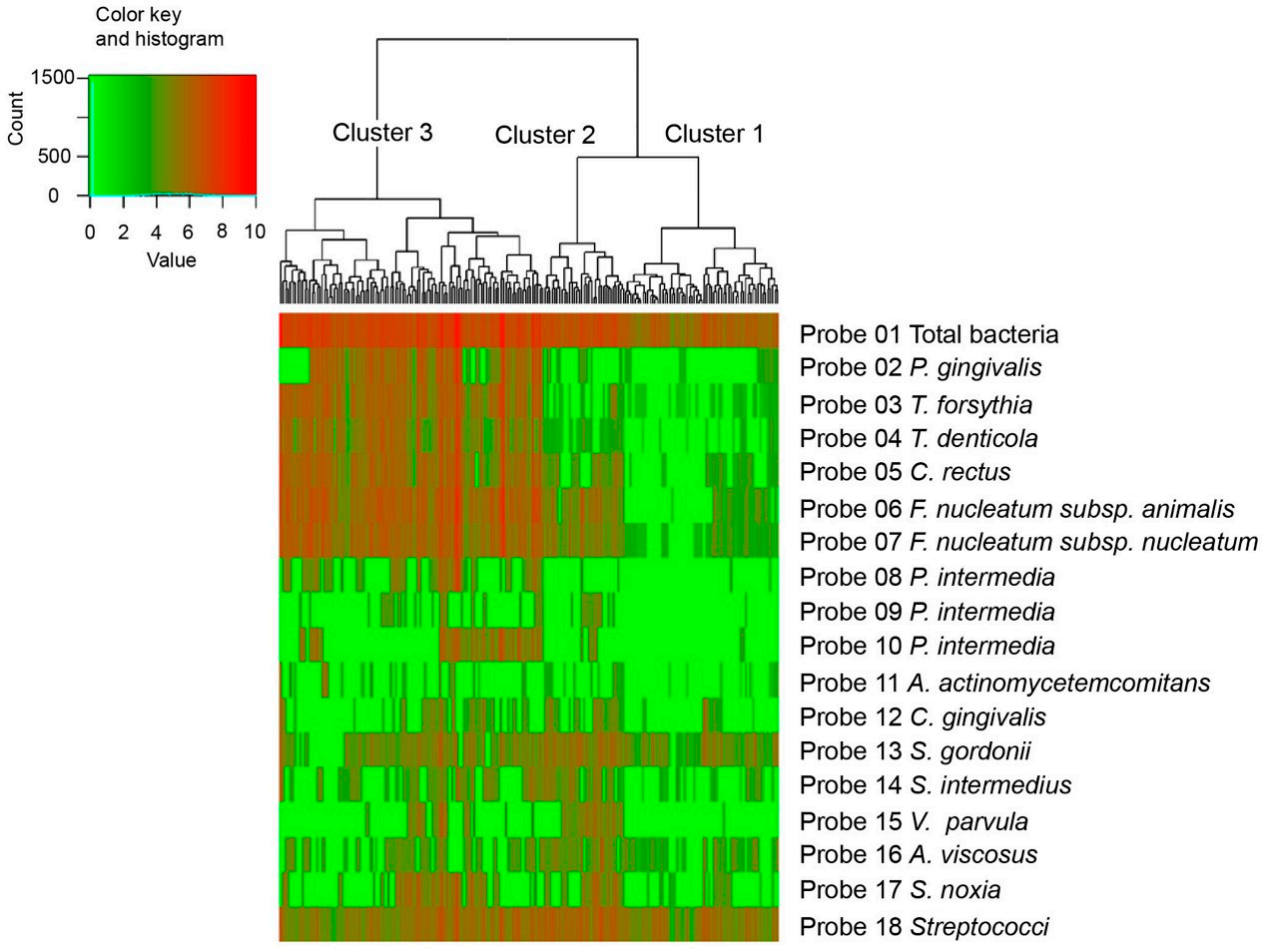
Heatmap of samples from patients before scaling and root planing (SRP). Cluster analysis was used to classify 204 samples into three clusters, based only on the quantities of 17 species of bacteria (counts per one paper point, presented as log10 values). When the number of bacteria was 0, it was treated as 0.1.

Cluster analysis was then performed to classify 132 samples obtained from patients at their first visit and 72 samples from healthy volunteers, based only on the quantities of 17 species of bacteria (Fig 2, Table 3). The clinical parameters value seen in Table 3 were calculated from the cluster constituent sample after classification and were not used for cluster analysis. The subgingival microbiota obtained from patients at their first visit was classified into at least three clusters according to the similarity of the quantities of bacteria. There was a significant difference in PD between cluster 1 and cluster 3, also, cluster 2 and cluster 3, but there was no significant difference between cluster 1 and cluster 2. The BOP-positive rate was significantly higher in cluster 3 than in clusters 1 and 2 in order. GI and PlI tended to be significantly higher in cluster 3 than in cluster 1 and 2 in order.

**Table 3:** Clinical parameters and oral bacterial counts among the three clusters.

	Bacterial counts												
		Cluster 1 (n = 63)			Cluster 2 (n = 33)			Cluster 3 (n = 108)			All samples (n = 204)		
Probe no.	Probe	Log10 mean		Ratio^c^	Log10 mean		Ratio^c^	Log10 mean		Ratio^c^	Log10 mean		Ratio^c^
01	Total number of bacteria^d^	5.9	(0.1)	100%	6.7	(0.1)	100%	7.2	(0.1)	100%	6.7	(0.1)	100%
02	*P*. *gingivalis*^a^	0.9	(0.2)	0.2%	1.2	(0.3)	0.1%	4.7	(0.2)	5.5%	3.0	(0.2)	3.0%
03	*T*. *forsythia*^a^	1.5	(0.2)	0.1%	1.8	(0.3)	0.1%	5.7	(0.1)	6.4%	3.8	(0.2)	3.4%
04	*T*. *denticola*^d^	0.8	(0.2)	0.1%	2.7	(0.2)	0.1%	5.2	(0.1)	3.5%	3.4	(0.2)	1.9%
05	*C*. *rectus*^d^	1.5	(0.2)	0.2%	3.3	(0.3)	0.8%	5.5	(0.1)	4.7%	3.9	(0.2)	2.7%
06	*F*. *nucleatum subsp*. *animalis*^d^	1.6	(0.2)	0.6%	4.8	(0.2)	3.6%	6.0	(0.1)	12.0%	4.5	(0.2)	7.1%
07	*F*. *nucleatum subsp*. *nucleatum*^d^	2.5	(0.2)	0.4%	4.7	(0.1)	2.8%	5.8	(0.1)	7.6%	4.6	(0.1)	4.6%
08	*P*. *intermedia*^a^	0.2	(0.1)	0.0%	0.3	(0.2)	0.0%	2.8	(0.2)	0.5%	1.6	(0.2)	0.2%
09	*P*. *nigrescens*^e^	0.2	(0.1)	0.0%	1.6	(0.4)	0.1%	1.2	(0.2)	0.0%	0.9	(0.1)	0.0%
10	*S*. *constellatus*^a^	0.2	(0.1)	0.1%	0.8	(0.3)	0.3%	2.6	(0.3)	1.1%	1.6	(0.2)	0.6%
11	*A*. *actinomycetemcomitans*	0.5	(0.1)	0.0%	1.4	(0.3)	0.0%	1.0	(0.2)	0.1%	0.9	(0.1)	0.0%
12	*C*. *gingivalis*^e^	0.7	(0.2)	0.3%	3.2	(0.3)	0.4%	1.8	(0.2)	0.2%	1.7	(0.1)	0.2%
13	*S*. *gordonii*	4.1	(0.1)	4.4%	5.2	(0.1)	5.3%	3.8	(0.2)	0.7%	4.1	(0.1)	2.6%
14	*S*. *intermedius*^d^	1.2	(0.2)	0.3%	4.0	(0.3)	1.3%	2.5	(0.2)	0.5%	2.4	(0.1)	0.6%
15	*V*. *parvula*^d^	0.1	(0)	0.0%	3.8	(0.3)	1.8%	1.1	(0.2)	0.2%	1.3	(0.1)	0.4%
16	*A*. *viscosus*	2.6	(0.2)	1.1%	4.1	(0.2)	1.3%	2.5	(0.2)	0.2%	2.8	(0.1)	0.6%
17	*S*. *noxia*^e^	1.2	(0.2)	0.4%	3.5	(0.4)	2.4%	2.5	(0.2)	1.2%	2.3	(0.2)	1.1%
18	Streptococci	5.0	(0.1)	18.8%	5.9	(0.1)	16.4%	5.2	(0.1)	3.1%	5.3	(0.1)	10.1%
	Clinical parameters calculated from sample of configuring a cluster												
	PD (mm)^a^	2.9 (0.1)			3.4 (0.2)			6.2 (0.2)			4.7 (0.2)		
	BOP (positive rate)^b^	10%			21%			71%			44%		
		0	1	2	0	1	2	0	1	2	0	1	2
	GI (sites) ^b^	48	14	1	21	10	2	5	57	46	74	81	49
	PlI (sites) ^b^	45	18	0	10	17	6	8	67	33	63	102	39

The value in parentheses indicates standard error.

^a^P-value < 0.05 by Steel-Dwass test between clusters 1 and 3, also, between clusters 2 and 3. For bacterial counts, the p-value was adjusted based on the Bonferroni test.

^b^P-value < 0.05 by χ^2^ test for BOP, GI, and plI in any combination of the three clusters.

^c^The value of the ratio indicates the average percentage among total numbers of bacteria.

^d^P-value < 0.05 by Steel-Dwass test with the combination of all three clusters

^e^P-value < 0.05 by Steel-Dwass test adjusted by the Bonferroni test between clusters 1 and 2 and also between clusters 1 and 3.

PD, probing depth; BOP, bleeding on probing; GI, gingival index; plI, plaque index

Analysis of the characteristics of microbiota showed that there was a significant difference in the number of total bacteria, *T*.*denticola*, *C*.*rectus*, *F*. *nucleatum*, and *Streptococcus intermedia* in any combination of the three clusters. It also revealed that the proportion of *T*. *denticola*, *Campylobacter rectus*, and *F*. *nucleatum* to the total number of bacteria was particularly higher in cluster 3. Meanwhile, the numbers of *P*. *gingivalis*, *T*. *forsythi*a, *P*. *intermedia*, and *S*. *constellatus* were significantly different among clusters 1 or 2 vs. 3, and the proportion of these bacteria to the total number of bacteria was particularly high in cluster 3.

Overall, cluster 1 showed healthy profiles, with a mean PD of 2.9 mm and low amount of *P*. *gingivalis*, *T*. *forsythia*, and *T*. *denticola*. Cluster 2 showed an early-stage periodontal disease profile with a mean PD of 3.4 mm. The number of bacteria in cluster 2 was higher than that in cluster 1 for all species tested in this study. Cluster 3 showed an advanced-stage periodontal disease profile with a mean PD of 6.2 mm and a highest amount of *P*. *gingivalis*, *T*. *forsythia*, and *T*. *denticola*.

### Changes in microbiota after periodontal treatment

Subgingival plaque samples before and after SRP (n = 62) were compared using the Oral Care Chip. The clinical findings of after-SRP sites were characterized by a reduction in clinical parameters, which indicate the presence of inflammation (Table 4). The low p-values for bacterial count data indicated a correlation between clinical parameters and microbiota. In contrast, the change in microbiota showed a similar pattern to that observed for healthy sites after periodontal disease treatment, and the abundance ratio of some strains including streptococci was increased.

**Table 4:** Changes in the clinical parameters and oral bacteria in a 62-sample set following scaling and root planing (SRP)^a^.

	**Clinical parameters**							
		Before SRP			After SRP			P-value
	PD (mm)^a^	6.0 (0.3)			3.5 (0.2)			2.5 ⋅ 10^−9^
	BOP (positive rate)^b^	63%			23%			1.5 ⋅ 10^−6^
		0	1	2	0	1	2	
	GI (sites)^b^	3	34	25	43	18	1	8.0 ⋅ 10^−11^
	PlI (sites)^b^	4	41	17	43	16	3	9.3 ⋅ 10^−9^
	**Bacterial counts**							
Probe no.	Probe	Log10 mean		Ratio^c^	Log10 mean		Ratio^c^	P-value
01	Total number of bacteria^a^	7.0	(0.1)	100%	5.8	(0.1)	100%	6.4 ⋅ 10^−9^
02	*P*. *gingivalis*^a^	3.8	(0.4)	3.9%	1.3	(0.3)	1.6%	1.3 ⋅ 10^−8^
03	*T*. *forsythia*^a^	5.0	(0.2)	5.2%	2.1	(0.3)	0.9%	2.5 ⋅ 10^−9^
04	*T*. *denticola*^a^	4.5	(0.2)	2.8%	2.1	(0.3)	1.2%	8.4 ⋅ 10^−8^
05	*C*. *rectus*^a^	5.0	(0.2)	4.5%	2.4	(0.3)	1.6%	1.7 ⋅ 10^−8^
06	*F*. *nucleatum subsp*. *animalis*^a^	5.5	(0.2)	8.5%	3.1	(0.3)	2.8%	2.4 ⋅ 10^−8^
07	*F*. *nucleatum subsp*. *nucleatum*^a^	5.5	(0.1)	7.5%	3.2	(0.2)	2.5%	7.4 ⋅ 10^−9^
08	*P*. *intermedia*^a^	2.1	(0.3)	0.4%	0.9	(0.2)	0.3%	8.5 ⋅ 10^−4^
09	*P*. *nigrescens*	0.9	(0.2)	0.0%	0.6	(0.2)	0.0%	8.6 ⋅ 10^−1^
10	*S*. *constellatus* ^a^	2.3	(0.3)	0.9%	0.8	(0.2)	1.0%	2.4 ⋅ 10^−5^
11	*A*. *actinomycetemcomitans*	1.0	(0.2)	0.1%	0.5	(0.1)	0.0%	9.8 ⋅ 10^−1^
12	*C*. *gingivalis*	2.0	(0.3)	0.2%	1.7	(0.2)	0.2%	1.0
13	*S*. *gordonii*	4.0	(0.2)	2.0%	3.4	(0.2)	3.8%	2.6 ⋅ 10^−1^
14	*S*. *intermedius*	2.3	(0.3)	1.0%	2.0	(0.3)	1.0%	1.0
15	*V*. *parvula*	1.1	(0.3)	0.5%	1.1	(0.2)	0.8%	1.0
16	*A*. *viscosus*	3.1	(0.2)	0.6%	3.0	(0.2)	2.0%	1.0
17	*S*. *noxia*	2.0	(0.3)	1.1%	1.7	(0.3)	1.2%	1.0
18	Streptococci^a^	5.3	(0.1)	6.5%	4.6	(0.1)	11.7%	1.0 ⋅ 10^−3^

The value in parentheses indicates standard error.

^a^ P-value < 0.05 by Wilcoxon’s signed rank test for PD and bacterial counts. For bacteria, the p-value was adjusted by a Bonferroni test.

^b^P-value < 0.05 by McNemar test for BOP, GI, and plI.

^c^The value of the ratio indicates the average percentage among total numbers of bacteria.

PD, probing depth; BOP, bleeding on probing; GI, gingival index; plI, plaque index

## Discussion

A previous study [17] showed that poor oral hygiene increases the amount of dental plaque (bacterial plaque) that attaches to the surface of teeth and changes the composition of subgingival microbiota, leading to inflammation in the gingiva. Hence, bacteria were confirmed to be the major cause of periodontal disease. Especially, *P*. *gingivalis*, *T*. *forsythia*, and *T*. *denticola* of the red complex, a group of bacteria that are frequently isolated from deep periodontal pockets in patients with periodontal disease were considered responsible for the initiation and progress of periodontal disease [2]. Studies made from the view point of dysbiosis have increased along with the progress of microbiota analysis technology. It is hypothesized that periodontal disease is caused by the interactions between the host and the dysbiotic subgingival microbiota recently [4].

To monitor these microbiota changes and confirm their association with periodontal disease, simultaneous and quantitative detection of multiple bacteria that make up the subgingival microbiota is necessary. Existing methods for analysis of microbiota data are not quantitative or clinically applicable. Recently, a new method for simultaneous multiple bacteria detection has been developed [10]. In this study, we have demonstrated that the use of Oral Care Chip was quantitative as shown in the comparison with real-time PCR. This method can easily measure multiple bacterial species at the same time in a clinically applicable way.

Using this method, we have shown for the first time, to the best of our knowledge, the composition monitoring of subgingival microbiota in large-scale samples linking it with the PD and BOP obtained. This study demonstrated that the subgingival microbiota obtained from patients at their first visit can be classified into at least three clusters based on similarities in the number of each bacterium: these results indicated that the similarities in the number of each bacterium are associated with clinical findings, and that bacterial testing to diagnose periodontal disease was effective. The observations that samples with severe periodontal disease were enriched in cluster 3 and that those with moderate periodontal disease were enriched in cluster 2 indicated that the numbers of total bacteria, *T*. *denticola*, *C*. *rectus*, and *F*. *nucleatum* have gradually increased from the stage before PD deepens because there was a significant difference in any combination of the three clusters. Conversely, *P*. *gingivalis* and *T*. *forsythia*, *P*. *intermedia*, and *S*. *constellatus* were expected to increase after PD became deep to some extent because they were significantly higher in cluster 3 than in clusters 1 and 2. Hence, these changes in the microbiota can predict the progress of periodontal tissue destruction [18]. Moreover, a comparison of cluster 1 and 2 revealed that microbiota patterns were different even in the samples with no statistical difference in PD. Therefore, it is possible to determine the detailed condition of early periodontal disease by measuring the microbiota of a sample before PD grows deeper. From another point of view, 82 of 204 samples obtained at the first time visit had a PD of 6 mm or greater, and 81% of the 82 samples were found to have *P*. *gingivalis* (S1 File). This result is consistent with the Meta-analysis report which summarized studies from several countries including Japan which reported that *P*. *gingivalis* detective rate was 78% [4].

In this study, most sites treated for periodontal disease showed a remarkable improvement in clinical findings and a decrease in many target bacteria including the total bacteria. The similarity of these microbiota profiles between after-treatment samples and clinically healthy samples obtained at the first visit indicates that the subgingival microbiota after periodontal treatment changed to a state close to that of healthy sample. Oppositely, as for the two sites where the total number of bacteria increased exceptionally after treatment, one site was the only sample with deeper PD and worsened BOP after treatment. The other test site showed no change before and after treatment in the clinical findings of PD and BOP. The Oral Care Chip enables precise analyses of changes in the microbiota and is also effective to study the profiles of microbiota found in sites with no improvements in clinical symptoms after treatment.

The main limitation of this study was that the number of samples was insufficient, therefore the periodontal disease threshold used as a test result could not be defined as the absolute number of bacteria. Acquiring clinical and bacterial data of the same subject over time can be beneficial. Future research will aim to clarify the changes in the microbiota by following the rate of progress of the disease at one site, the effects of age, sex, and the consumption of antibiotics.

Our findings indicate that an increase in the ratio of *C*. *rectus* and *F*. *nucleatum* in the subgingival microbiota, followed by the emergence of the red complex, can be a predictor of the progress of periodontal tissue destruction. These results demonstrate that this novel bacterial detection method using the newly developed Oral Care chip is effective to identify dysbiosis in the mouth. This novel bacterial detection platform might also be useful not only for the analysis of oral microbiota but also for the analysis of intestinal microbiota, skin microbiota, and environmental microbiotas by modifying the design of the DNA probes used.

## Supporting information

S1 File Oral Care Chip data and real-time PCR data. (XLSX)

S2 File (XLSX)

S1 Fig Schematic diagram of quantification using the Oral Care Chip. (A) Design of control DNA. (B) Genomic DNA in a sample and control DNA are amplified using common universal primers by competitive PCR. An amplicon having a complementary strand with two probes is distributed to the two probes at a constant rate upon hybridization. (C) The ratio is unique for each probe and defined as the hybrid coefficient. These were calculated in advance experimentally (S1 Fig). Analysis of signal intensity after hybridization was performed in two steps. For the first step, the total number of bacteria was calculated from the SI of competitive PCR products. In the second step, the number of each species of bacteria was calculated by multiplying the SI ratio specific for each probe and the total number of bacteria. To correct for the binding capacity of each specific probe, the SI of each probe was corrected using the hybridization coefficient as described above (S3 Fig).
(TIF)

S2 Fig Standard curve of the probe for total number of bacteria generated by competitive PCR. This curve was used to determine the molecular weight of the bacterial genome from the signal intensity (SI) obtained after competitive PCR. (A) Standard curve generated from triplicate analyses. For the analyses, MSA-1003^™^ (2.9 × 10^−3^ to 190 amol of 16S rRNA), and 0.50 amol of control DNA were amplified by competitive PCR assays. (B) Oral Care Chip images of template DNA (2.9 × 10^−3^, 0.73, or 190 amol).
(TIF)

S3 Fig Signal intensity (SI) ratio of bacterium-specific probe to probe for the total number of bacteria. The data shown are SI values obtained by individually hybridizing DNA purified after PCR amplification from a specific plasmid (Table 1), from approximately 16 to 1000 fmol of 16S rRNA, to the Oral Care Chip once. The slope indicates the hybridization coefficient of each probe. Shown are data for: (A) Probe no.2; (B) Probe no.3; (C) Probe no.4; (D) Probe no.5; (E) Probe no.6; (F) Probe no.7; (G) Probe no.8; (H) Probe no.9; (I) Probe no.10; (J) Probe no.11; (K) Probe no.12; (L) Probe no.13; (M) Probe no.14; (N) Probe no.15; (O) Probe no.16; (P) Probe no.17; (Q) Probe no.18. When individual probes were evaluated, PCR products from plasmid DNA as a template was purified using the MinElute PCR purification kit (Qiagen, Hilden, Germany) and suspended in a hybridization solution. The plasmid DNA with the appropriate 16S rRNA sequence (sequence accession numbers are given in Table 1) was inserted into pUC19 (FASMAC, Kanagawa, Japan). The reason for purifying the amplified product after PCR was to exclude extra primers and to calculate the number of moles from the DNA concentration. To compare the utility of each probe, the molar concentrations of the template DNA were set based on conditions.
(TIF)

S4 Fig Age at first visit of subjects with sample and PD. The sample size is 204.
(TIF)

S5 Fig Tooth notation on all teeth and PD at first visit. The sample size is 204.
(TIF)

S1 Table Sequences of control DNA and probe. (DOCX)

S2 Table Sequences of primers and probes^a^ used in real-time PCR. Probe sequences are provided in parentheses. FAM, carboxy fluorescein; TAMRA, tetramethyl-6-carboxyrhodamine.
(DOCX)
