# Supplementary figures and images for: Development of Oral Care Chip, a novel device for quantitative detection of the oral microbiota associated with periodontal disease
Source: PLoS One. 2020 Feb 28;15(2):e0229485. doi: 10.1371/journal.pone.0229485 (PMC7048280; doi:10.1371/journal.pone.0229485)

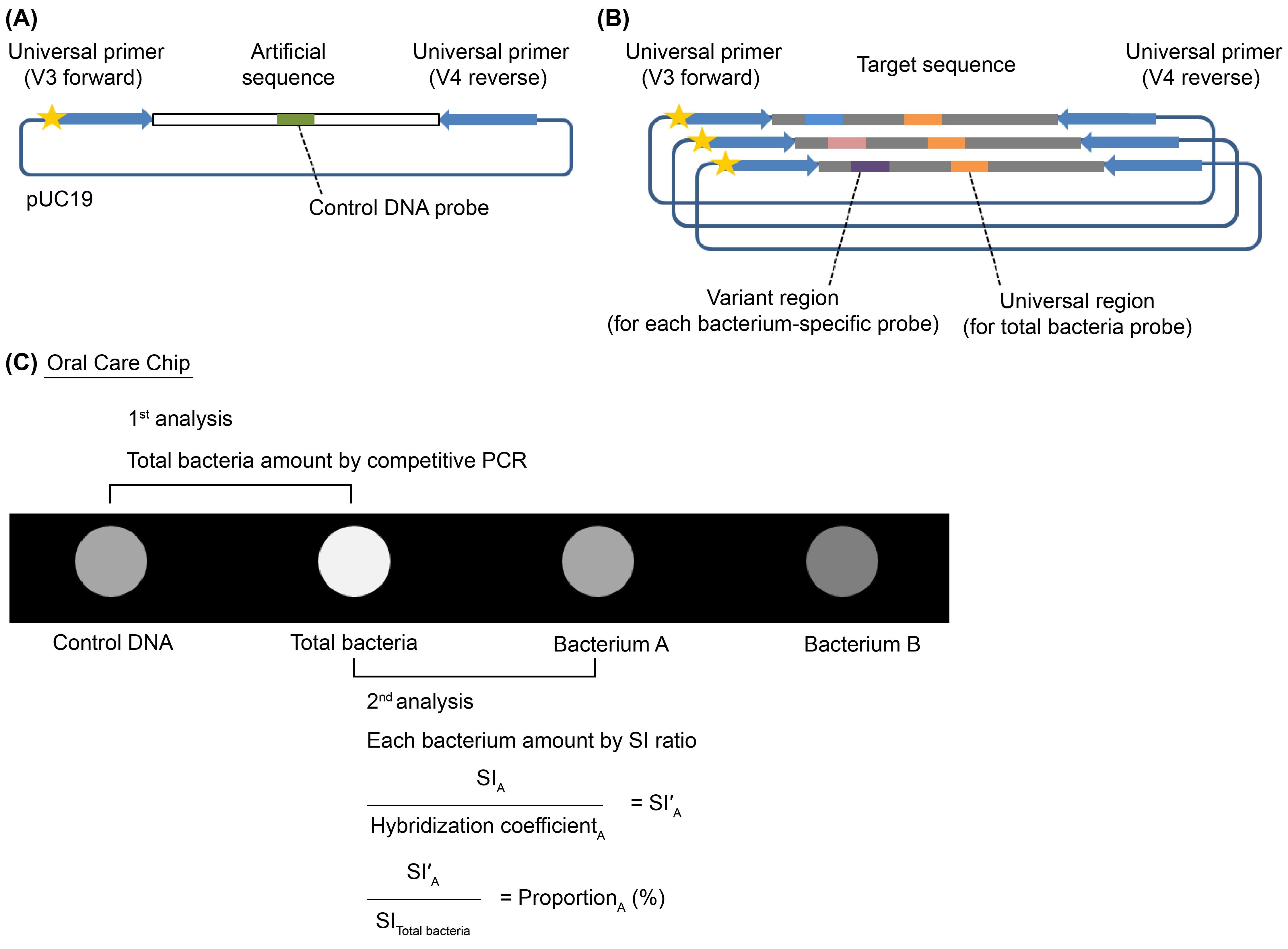

Supplement: S1 Fig — (A) Design of control DNA. (B) Genomic DNA in a sample and control DNA are amplified using common universal primers by competitive PCR. An amplicon having a complementary strand with two probes is distributed to the two probes at a constant rate upon hybridization. (C) The ratio is unique for each probe and defined as the hybrid coefficient. These were calculated in advance experimentally (S1 Fig). Analysis of signal intensity after hybridization was performed in two steps. For the first step, the total number of bacteria was calculated from the SI of competitive PCR products. In the second step, the number of each species of bacteria was calculated by multiplying the SI ratio specific for each probe and the total number of bacteria. To correct for the binding capacity of each specific probe, the SI of each probe was corrected using the hybridization coefficient as described above (S3 Fig). (TIF) [file pone.0229485.s003.tif]

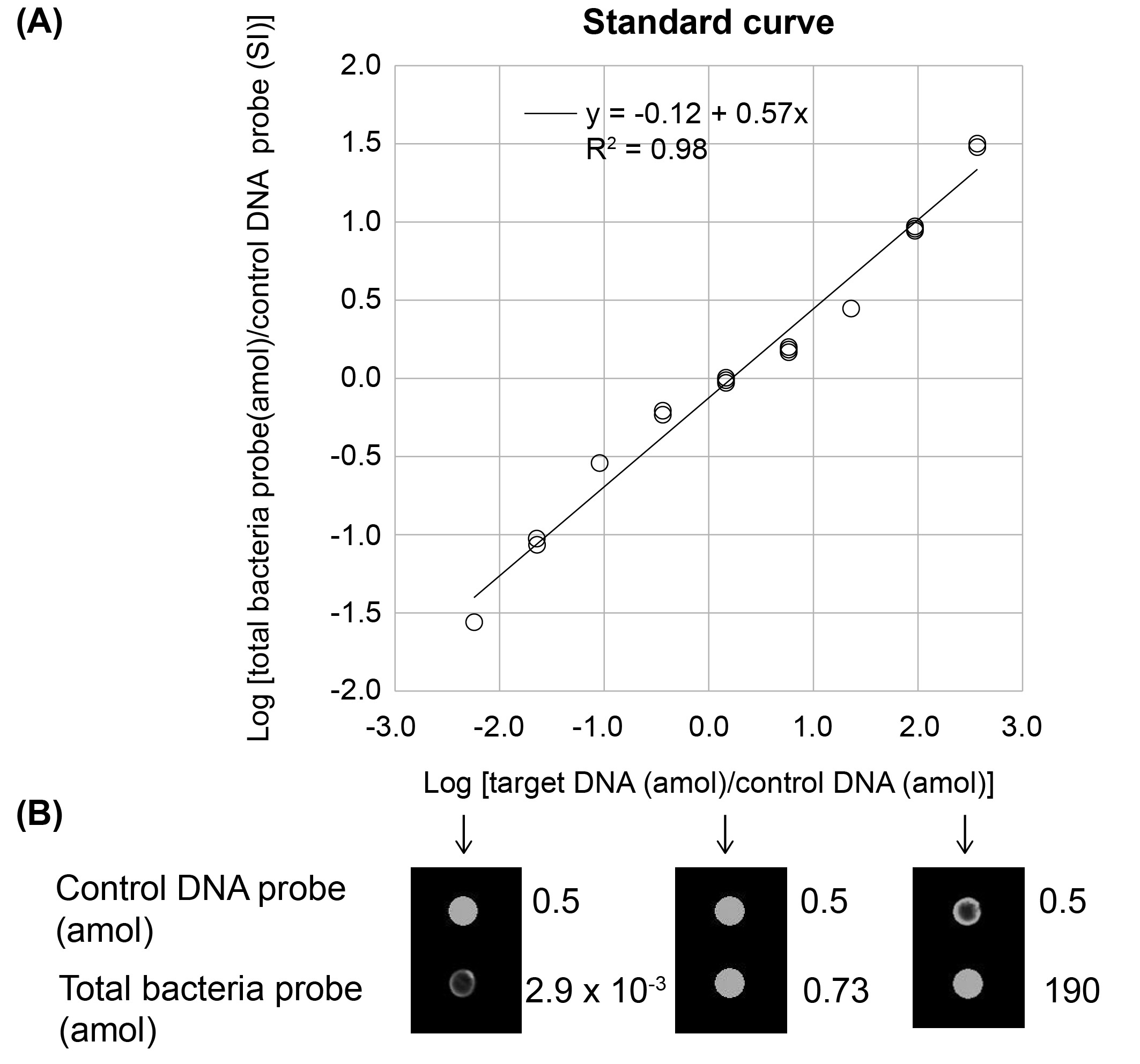

Supplement: S2 Fig — This curve was used to determine the molecular weight of the bacterial genome from the signal intensity (SI) obtained after competitive PCR. (A) Standard curve generated from triplicate analyses. For the analyses, MSA-1003™ (2.9 × 10^−3^ to 190 amol of 16S rRNA), and 0.50 amol of control DNA were amplified by competitive PCR assays. (B) Oral Care Chip images of template DNA (2.9 × 10^−3^, 0.73, or 190 amol). (TIF) [file pone.0229485.s004.tif]

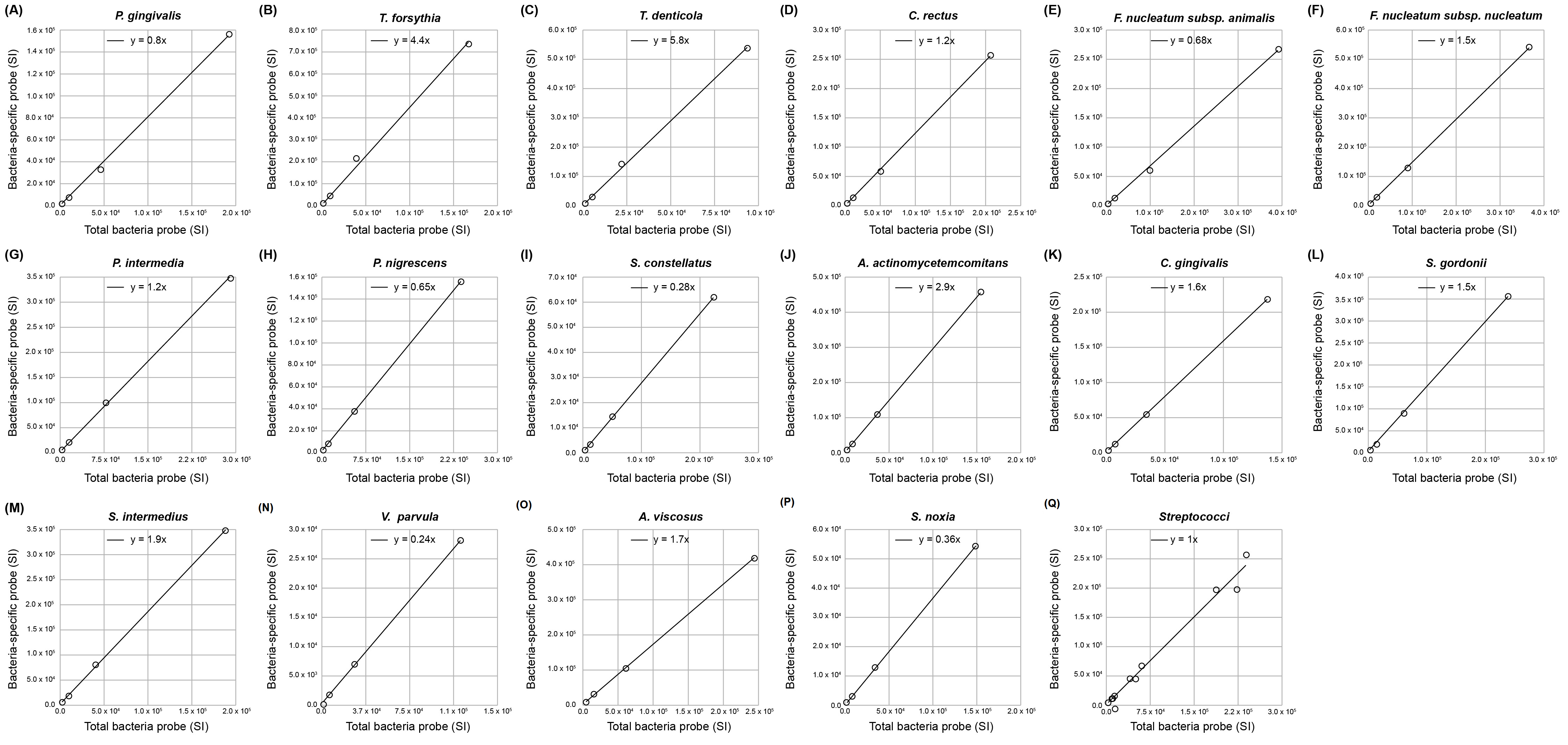

Supplement: S3 Fig — The data shown are SI values obtained by individually hybridizing DNA purified after PCR amplification from a specific plasmid (Table 1), from approximately 16 to 1000 fmol of 16S rRNA, to the Oral Care Chip once. The slope indicates the hybridization coefficient of each probe. Shown are data for: (A) Probe no.2; (B) Probe no.3; (C) Probe no.4; (D) Probe no.5; (E) Probe no.6; (F) Probe no.7; (G) Probe no.8; (H) Probe no.9; (I) Probe no.10; (J) Probe no.11; (K) Probe no.12; (L) Probe no.13; (M) Probe no.14; (N) Probe no.15; (O) Probe no.16; (P) Probe no.17; (Q) Probe no.18. When individual probes were evaluated, PCR products from plasmid DNA as a template was purified using the MinElute PCR purification kit (Qiagen, Hilden, Germany) and suspended in a hybridization solution. The plasmid DNA with the appropriate 16S rRNA sequence (sequence accession numbers are given in Table 1) was inserted into pUC19 (FASMAC, Kanagawa, Japan). The reason for purifying the amplified product after PCR was to exclude extra primers and to calculate the number of moles from the DNA concentration. To compare the utility of each probe, the molar concentrations of the template DNA were set based on conditions. (TIF) [file pone.0229485.s005.tif]

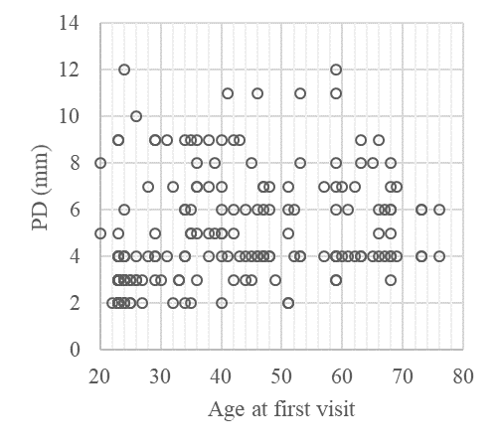

Supplement: S4 Fig — The sample size is 204. (TIF) [file pone.0229485.s006.tif]

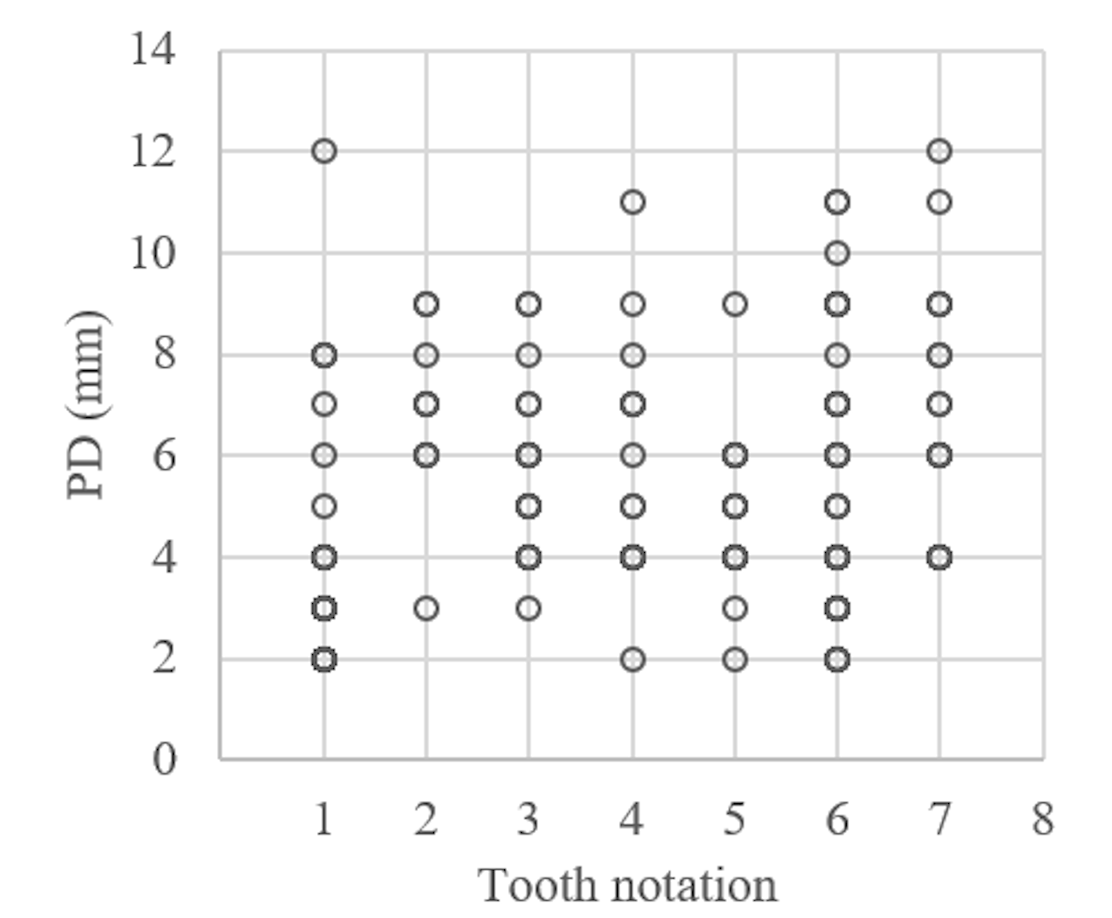

Supplement: S5 Fig — The sample size is 204. (TIF) [file pone.0229485.s007.tif]
