# Supplementary material for: Development of Oral Care Chip, a novel device for quantitative detection of the oral microbiota associated with periodontal disease
Source: PLoS One. 2020 Feb 28;15(2):e0229485. doi: 10.1371/journal.pone.0229485 (PMC7048280; doi:10.1371/journal.pone.0229485)
Supplement: S1 Table — (DOCX) [file pone.0229485.s008.docx]

**S1 Table. Sequences of control DNA and probe.**

| DNA | Sequence (5′–3′) |
| --- | --- |
| Control DNA | GTTCCTACGGGAGGCAGCAGTAGGGCTAAGACAAACGCTAACGGTACACCCTAGATGGGAGCTTGTAGCTAGATCGCTAAGTCCTACCGACATGTAGGCATACTCACGAAGGCAATTCCCTGAAAGCCTCGTCTTATCCCGAACTTGGCATCTGCTGATACGTCAGGTTGAACGCGTACATTTACCTGTCATGCGTGGGCCTTCTCCGAATAGCCTACGTAGTGATATCGCTGGTCGAATAGGCGGATTGCTCATAAATGCACATTGGCTAAGGCCCACGGAACACGAATCACGTGAGATCACTTACTATTCGACGGAACTACTATACGCACCGGGACATGCAAGTAGCGTCCCACAAGCATAAGGAACTCTATACTCGCCATCTACGCAGCTACAGGGGATACACGTATGAGCGGTTACGAAGTAAAGGGTAGCAACAGGATTAGATACCCTGGTAGTCCAC |
| Control DNA probe | CTATTCGACCAGCGATATCACTACGTAGGC |
