# Supplementary material for: Development of Oral Care Chip, a novel device for quantitative detection of the oral microbiota associated with periodontal disease
Source: PLoS One. 2020 Feb 28;15(2):e0229485. doi: 10.1371/journal.pone.0229485 (PMC7048280; doi:10.1371/journal.pone.0229485)
Supplement: S2 Table — Probe sequences are provided in parentheses. FAM, carboxy fluorescein; TAMRA, tetramethyl-6-carboxyrhodamine. (DOCX) [file pone.0229485.s009.docx]

**S2 Table. Sequences of primers and probes**^a^ **used in real-time PCR.**

Probe sequences are provided in parentheses. FAM, carboxy fluorescein; TAMRA, tetramethyl-6-carboxyrhodamine.

| **Target** | **Sequence (5′–3′)** | **Product size (bp)** | **Reference** |
| --- | --- | --- | --- |
|  | TCGGTAAGTCAGCGGTGAAAC |  |  |
| ***P. gingivalis*** | GCAAGCTGCCTTCGCAAT | 132 | (15) |
|  | (FAM-CTCAACGTTCAGCCTGCCGTTGAAA-TAMRA) |  |  |
|  | GGGTGAGTAACGCGTATGTAACCT |  |  |
| ***T. forsythia*** | CCCATCCGCAACCAATAAA | 108 | (14) |
|  | (FAM-CCCGCAACAGAGGGATAACCCGG-TAMRA) |  |  |
|  | CCGAATGTGCTCATTTACATAA |  |  |
| ***T. denticola*** | GATACCCATCGTTGCCTTGGT | 101 | (12) |
|  | (FAM-ATGGGCCCGCGTCCCATTAGC-TAMRA) |  |  |
|  | TCCACCGATGAATCTTTGGTC |  |  |
| ***Prevotella intermedia*** | ATCCAACCTTCCCTCCACTC | 82 | (12) |
|  | (FAM-CGTCAGATGCCATATGTGGACAACATCG-TAMRA) |  |  |
|  | CTTACCTACTCTTGACATCCGAA |  |  |
| ***A. actinomycetemcomitans*** | ATGCAGCACCTGTCTCAAAGC | 56 | (13) |
|  | (FAM-AGAACTCAGAGATGGGTTTGTGCCTTAG-TAMRA) |  |  |
|  | TCCTACGGGAGGCAGCAGT |  |  |
| **All bacteria** | GGACTACCAGGGTATCTAATCCTGTT | 441 | (12) |
|  | (FAM-CGTATTACCGCGGCTGCTGGCAC-TAMRA) |  |  |
